# Bacterial extracellular vesicles in the brain: Pathological effects and therapeutic possibilities

**DOI:** 10.4103/NRR.NRR-D-25-00236

**Published:** 2025-06-19

**Authors:** Yaiza M. Arenas, Paula Izquierdo-Altarejos, Gaspar Pérez-Martínez, Vicente Felipo, Marta Llansola

**Affiliations:** 1 Laboratory of Neurobiology, Centro de Investigación Príncipe Felipe (CIPF), Valencia, Spain; 2 Departamento de Patología, Facultad de Medicina, Universidad Valencia, Valencia, Spain; 3 INCLIVA Instituto de Investigación Sanitaria, Valencia, Spain; 4 Lactic acid bacteria and probiotics Laboratory, Instituto de Agroquímica y Tecnología de Alimentos, Spanish National Research Council (CSIC), Valencia, Spain

**Keywords:** bacteria, bacterial extracellular vesicles, gut–brain axis, inflammation, microbiota, neuroinflammation, neurological diseases, neurotransmission, pathogenic, probiotic, therapeutic treatment

## Abstract

The mechanisms leading to neurological and neurodegenerative diseases are not completely known, and new, more effective, therapeutic treatments are necessary for most neurological pathologies. The treatment of neurological and neurodegenerative diseases is complicated due to the blood–brain barrier, which makes it difficult for drugs to access the brain areas in which they must act to improve the pathology. A tool that can help to overcome this difficulty is the use of extracellular vesicles, which can easily cross the blood–brain barrier. The extracellular vesicles are considered a main way of communication between the brain and the rest of the body, with important implications for the physiopathology and therapy of neurological diseases. In recent years, the involvement of microbiota in many neurological pathologies, as well as its possible therapeutic role, has also become evident. A key mediator in the pathologic and beneficial effects of microbiota seems to be the bacterial extracellular vesicles. There is an important communication between the brain and the intestinal microbiota (the gut–brain axis), by which the microbiota influences brain function, impacts on mental health, and plays a role in different neurological and neurodegenerative diseases. The identification of the mechanisms involved in this gut–brain axis is essential to understanding the mechanisms of neurological pathologies and to developing more effective treatments for these diseases. Bacterial extracellular vesicles would play a relevant role in these processes. This review compiles the recent information and evidence on the role of bacterial extracellular vesicles in brain pathologies and on the therapeutic utility of bacterial extracellular vesicles in neurological and neurodegenerative diseases. One advantage of bacterial extracellular vesicles compared to extracellular vesicles derived from other cell types, such as stem cells, is that bacterial extracellular vesicles are generally easier to produce and modify. Bacterial extracellular vesicles may be easily modified to target a specific pathology and/or to enhance its therapeutic efficacy. Although the studies are still scarce, they open a wide field of possibilities for future studies, which will lead to a deeper understanding of the role of microbiota and bacterial extracellular vesicles in neurological pathologies and the underlying mechanisms, as well as to the development of new treatments based on the use of bacterial extracellular vesicles in neurological diseases.

## Introduction

Extracellular vesicles (EVs) are produced by all kingdoms of life, from mammalian cells to yeast, fungi, and bacteria. They are often thought to play a role in communication in living organisms, fitting the simplistic idea of a “message in a bottle.” In complex environments such as the human gut, bacterial extracellular vesicles (BEVs) could be very important players in the communication between bacteria, between bacteria and host cells, but also in receiving signals in the BEVs from the host. Therefore, a broader definition that includes their ecological and evolutionary connections in a living system would be “evolutionarily conserved intercellular communicasomes” (Kim et al., 2015).

As occurs in the case of EVs originating from eukaryotic cells, BEVs participate in the mediation of both pathological effects due to their involvement in the mechanisms by which bacteria contribute to diverse pathological conditions affecting distant organs and systems. Moreover, BEVs have also been implicated in the mechanisms of action of probiotics, which have been shown to improve health. The potential of BEVs in the mechanisms by which dietary probiotics exert beneficial actions on various pathologies supports the possibility of using them as therapeutic vehicles. This can be achieved by administering them after isolation from beneficial bacteria and even after modifying their characteristics and cargo to improve their therapeutic potency.

Since the microbiota largely mediates the close relationship between the intestine and the brain through the gut-brain axis, the formation and transport of BEVs may be a main contributor to the interaction between these two systems. Furthermore, the oral microbiome has also been shown to influence different neurological pathologies, and BEVs may also contribute to the mechanisms by which oral microbiota modulate cerebral function.

In this review, we summarize the evidence for the presence of BEVs in the nervous system, including the brain, as well as the pathological and beneficial effects of BEVs in various neuropsychiatric and neurodegenerative pathologies.

## Data Sources and Search Strategy

The literature search was conducted between October 2024 and January 2025 using the Google Scholar and PubMed electronic databases. Initially, no date restrictions were applied to select the most relevant publications. The search strategy utilized keywords and phrases, including “bacteria extracellular vesicles AND microbiota,” “bacteria extracellular vesicles AND probiotic,” and “bacteria extracellular vesicles AND gut-brain axis/ OR (neurological AND neurodegenerative disease)/ AND therapy.” The cited articles are original research publications, with the majority of referring research works published between 2020 and 2025, with some exceptions relating to timeless essential concepts. Bibliographies of related original research publications about bacterial extracellular vesicles in the nervous system and pathology and therapy were also searched for the main publications reporting scientific evidence of the discussion and conclusions of this review.

## Characteristics of Bacterial Extracellular Vesicles

**Figure 1:**
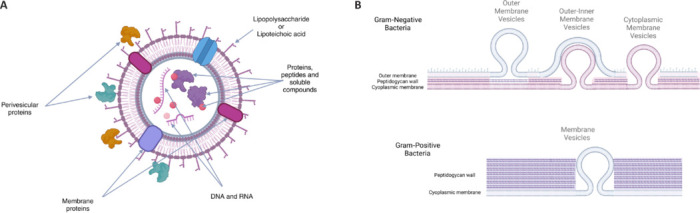
Bacterial extracellular vesicles. (A) In addition to membrane phospholipids and membrane proteins, bacterial extracellular vesicles contain cytoplasmic elements as cargo, such as proteins, peptides, DNA, RNA; they may also carry perivesicular proteins and possibly peptidoglycan fragments attached to the surface. (B) Genesis of bacterial membrane vesicles in gram-positive and gram-negative bacteria. The presence of a double membrane in Gran-negative bacteria suggests that there could be three possible types of vesicles, depending on their origin. Outer membrane vesicles, which are formed from the outer membrane and contain a cargo mainly with periplasmic elements; outer-inner membrane vesicles, which are formed by budding from the inner membrane but also protrude an outer membrane layer; and simple membrane vesicles or inner membrane vesicles, which contain only the inner membrane and a cytoplasmic cargo and are postulated to be formed by explosive lysis. Gram-positive bacteria have only an inner membrane and therefore secrete simpler membrane vesicles. Created with BioRender.com.

BEVs are very small, nanoparticle-sized (between 20–200 nm), spherical phospholipid membrane structures containing transmembrane proteins and lipopolysaccharides (LPSs) with a rich cargo of lipids, metabolites, nucleic acids, and cytoplasmic proteins, which can also be covered by perivesicular proteins (**Figure 1**). A very wide variety of functions have been proposed for BEVs, including cellular detoxification, transport of virulence factors, interception of bacteriophages, induction of eukaryotic host defense factors, bacterial communication, and horizontal gene transfer. BEVs were first described in Gram-negative bacteria, which have an inner and outer membrane containing the peptidoglycan cell wall and the vesicles are formed by blebbing of the outer membrane, therefore they were called outer membrane vesicles (OMVs), but in this bacterial group there is another type of cytoplasmic membrane vesicles, which are formed by the action of autolysins and originate from the inner cell membrane (Toyofuku et al., 2019). Different authors suggest that the cargo content of these vesicles would be different, as well as their possible functions (Toyofuku et al., 2023); this is because OMVs would be enriched in hydrophobic compounds and periplasmic proteins, whereas cytoplasmic membrane vesicles would be enriched in cytoplasmic contents, including RNAs and DNA (Toyofuku et al., 2019). However, there are very interesting facts that challenge this classification of BEVs, such as the transfer of virulence genes within OMVs by virulent strains of *Escherichia* (*E.*) *coli* (Kolling and Matthews, 1999) and by the presence of chromosomal DNA within OMVs of the Gram-negative *Pseudomonas aeruginosa* (Bitto et al., 2017). Other Gram-negative OMVs contain different amounts of genetic material, as DNAse-treated OMVs from *Aeromonas veronii, Enterobacter cloacae*, and *E. coli* were able to efficiently transfer plasmids across the species barrier (Tran and Boedicker, 2017). Indeed, these studies, together with studies on *Acinetobacter baumannii*, showed that *BEV* gene transfer could be a very important mechanism for the spread of antibiotic resistance genes (Fulsundar et al., 2019). As a consequence, in Gram-negative bacteria, a new BEV group has been defined as outer-inner membrane vesicles, which are possibly formed by blebbing of the inner membrane through a weakened cell wall, possibly by the action of endolysins (Macion et al., 2021). Since *Firmicutes* (Gram-positive bacteria) lack an outer membrane, BEV formation in these bacteria was initially explained by explosive cell lysis or bacterial cell senescence (Kim et al., 2015). However, a subsequent study has shown that BEV formation in the pathogen *Staphylococcus aureus* is a controlled process that requires of specific modulins that regulate cytoplasmic membrane budding and autolytic enzymes that facilitate passage through the cell wall (Wang et al., 2018). Furthermore, the efficiency of BEV production/export, different protein content of the cargo, and the biological activity depend on the environmental and growth conditions (Rodovalho et al., 2021; Lei et al., 2024).

It has been proposed that adhesins present on the surface of BEVs (i.e., perivesicular proteins) may recognize specific cell host receptors that facilitate the delivery of the vesicle cargo (Macion et al., 2021). BEVs carry DNA, RNA, lipoproteins, LPS, lipoteichoic acid, and peptidoglycan fragments, also known as microorganism-associated molecular patterns, which are recognized by pattern recognition receptors in host cells. BEVs may then simply fuse with the cell membrane in the cholesterol-rich lipid rafts (Kaparakis et al., 2010) or, most likely, they are taken up by clathrin/dynamin-mediated endocytosis-macropinnocytosis processes in the case of OMVs (Vanaja et al., 2016) as well as BEVs from Gram-positive bacteria (Champagne-Jorgensen et al., 2021a; da Silva et al., 2023).

### Biodistribution of bacterial extracellular vesicles

BEVs that circulate in the host bloodstream are mainly derived from gut microbes. In humans and mice, the amount of circulating BEVs is directly related to the permeability of the intestinal mucosa, and increases significantly with age and with health conditions that affect the intestinal barrier, such as colorectal cancer and colitis (Ou et al., 2023). Despite this, BEVs have been found in blood from healthy donors, in the absence of intestinal barrier disruption, and they seem interact mainly with monocytes (Schaack et al., 2022).

Currently, we know that the gut microbiome of healthy individuals is predominantly colonized by *Firmicutes* and *Bacteroidetes*. *Bacteroidetes*, as most Gram-negative bacteria, have LPS bound to the exterior of the outer membrane. LPSs have moderate to severe proinflammatory character and have been used for the selective identification of OMVs and BEVs in the gut, serum, and urine (Nah et al., 2019; Ricci et al., 2020). Rigorous comparisons have shown that BEVs found in urine are significantly biased compared to those found in serum, and proportions differ from those found in feces (Nah et al., 2019); however, urine is a readily accessible body fluid that can be obtained by minimally invasive procedures, and different authors have shown that proportions of BEVs in urine were sufficiently consistent to be used as markers for disease diagnosis (Park et al., 2021). To afford the analysis of possible biomarkers of BEV-host interaction it was performed a characterization of nanovesicles from *Lactobacillus johnsonii* N6.2 and the cell membrane protein named Sdp was selected as a possible biomarker. Sdp was detected after administration of these nanovesicles to *in vitro* human intestinal epithelial Caco-2 cell model. In addition, it was shown that volunteers developed IgA and IgG antibodies against nanovesicles and Sdp domains after oral administration (Harrison et al., 2021).

The presence of *in vitro* and *in vivo* administered BEVs in different cells, tissues, and organs of the host has been demonstrated (Kesty et al., 2004; Kuehn and Kesty, 2005; Jones et al., 2020). Internalization of *E. coli* BEVs with enterotoxin into intestinal epithelial cells and adrenal cells was shown to occur through cholesterol-rich lipid rafts, indicating that BEVs should be a vehicle to introduce enterotoxins into host cells (Kesty et al., 2004). BEVs from the major human gut commensal bacterium, *Bacteroides thetaiotaomicron*, were acquired by intestinal epithelial cells *in vitro* principally via dynamin-dependent endocytosis. Oral administration of these BEVs after fluorescence labelling allowed *in vivo* detection of BEVs in different tissues, accumulating mainly in the liver (Jones et al., 2020). Intravenously administered BEVs from *Bifidobacterium longum* and *Lactobacillus plantarum* WCFS1 mainly accumulated liver F4/80 cells and splenic CD169 macrophages. Subcutaneously administered BEVs accumulated in the lymph nodes and were mainly located in the B-lymphocyte zone, indicating that exogenously administered probiotic-derived EVs showed a similar biodistribution, irrespective of the EVs-secreting cell type (Morishita et al., 2023).

A previous study has demonstrated the delivery of cargoes by BEVs to human and animal cells (Kuehn and Kesty, 2005). It has been shown that *B. subtilis* BEVs can be transported to the basolateral space in Caco-2 cells, suggesting that this could be the first step allowing BEVs to reach the bloodstream for further delivery up to extraintestinal tissues and organs (Rubio et al., 2020). It has been proposed that if M cells in Peyer’s patches behaved similarly, endosome-enclosed BEVs could access the gut-associated lymphoid tissues (Macion et al., 2021). Bittel et al. (2021) showed the release of BEV content in intestinal stem cells and macrophages, as well as their distribution to different organs, using a modification in *E. coli* OMVs. *E. coli* was engineered to express Cre-recombinase into mice with a *Rosa26.tdTomato*-reporter background, which leveraged the Cre-LoxP system to report the transfer of bacterial OMVs to recipient cells *in vivo*.

### Extracellular vesicles from bacteria in the brain

**Figure 2:**
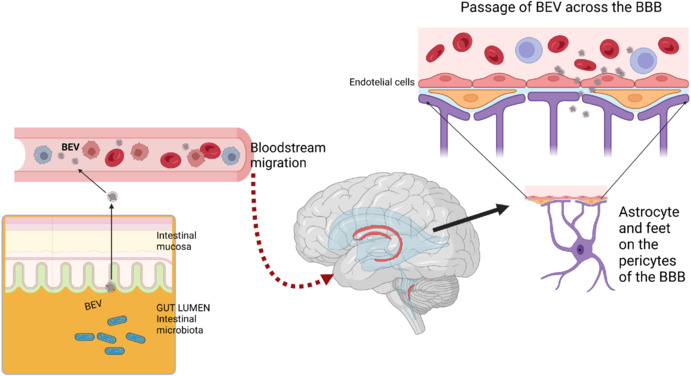
Possible migration pathway of gut bacterial extracellular vesicles to the central nervous system. Extracellular vesicles derived from gut bacteria can translocate across the epithelium by active transcitosis and then be transferred through other intestinal mucosal layers to the blood vessels by as yet unknown mechanisms. In the bloodstream, they could be transported to other sites in the body, including the brain, where bacterial extracellular vesicles could cross the blood–brain barrier through the interstitial spaces, but can also be incorporated into endothelial cells, to finally access different cell types into the brain. Created with BioRender.com. BBB: Blood–brain barrier; BEV: bacterial extracellular vesicle.

Some studies have also demonstrated the presence of BEVs in the brain. Pioneeringly, Wispelwey et al. (1989) hypothesized that the LPS of *Haemophilus influenzae* type b found in the cerebrospinal fluid after infection could be present as part of the OMVs of the bacteria. These authors demonstrated that the inoculation of OMVs has an equal or greater effect than LPS, altering the blood–brain barrier (BBB) and causing cerebrovascular inflammation. A systematic review of the supporting studies for the presence of BEVs in the brain was carried out by Kaisanlahti et al. (2023). Most studies have been performed in mouse models of Alzheimer’s disease (AD), ischemic stroke, stress, and periodontitis, since one way the microbiota influences the central nervous system is through infections and bacteria present in the oral cavity. The BEVs analyzed are derived mainly from the pathogens *Helicobacter Pilori*, *Salmonella*, *E. coli*, *Porphyromonas gingivalis*, and *Aggregatibacter actinomycetemcomitans*. However, the crossing of BBB by BEVs from *Lactobacillus plantarum, Bacillus subtilis*, and *Akkermansia muciniphila* has also been analyzed for a possible therapeutic application in stress (Kaisanlahti et al., 2023). Modasia et al. (2023) showed the presence of commensal *Bacteroides thetaiotaomicron*-derived BEVs in the central nervous system after intravenous administration in mice *in vivo* and then analyzed in several *in vitro* models how these BEVs cross the intestinal epithelium and the BBB. BEVs were acquired by both microglia and immature neuronal cells and induced microglial activation. A more recent study by Mottawea et al. (2025), performing metabolomics and proteomics analysis, shows that various neuronal-specific compounds are encapsulated within microbiota-derived BEVs from stools, such as arachidonyl-dopamine, gabapentin, glutamate, N-acylethanolamines, and an enrichment of enzymes involved in neurotransmission, mainly in the glutamine/glutamate/gamma-aminobutyric acid (GABA) pathway. These neuro-related proteins and metabolites were correlated with Bacteroides. Further study showed that *Bacteroides finegoldii* released BEVs with a high GABA content. Additionally, Mottawea et al. (2025) show that these BEVs cross BBB in an *in vitro* assay and that were biodistributed across mice organs, including the brain, liver, stomach, and spleen *in vivo*. This supports that BEVs have effects on neuronal function and neurotransmission when they reach the brain (**Figure 2**).

One of the main pathways of potential communication between the gut microbiota and the brain is the autonomic nervous system, which includes the enteric nervous system and the vagus nerve (Cuesta et al., 2021). To explore whether the pathogenic effects of the *Paenalcaligenes hominis*-derived EVs were mediated by the vagus nerve, Lee et al. (2020) administered the EVs to mice with and without celiac vagotomy. They found that vagotomy significantly reduced the cognitive impairment and the neuroinflammation induced by these EVs, suggesting that they may be transported into the brain by the vagus nerve. The authors also showed that vagotomy prevents the infiltration of the EVs into the hippocampus (Lee et al., 2020).

### Pathological Effects of Bacterial Extracellular Vesicles

From a clinical standpoint, bacteria are considered “germs” and any residual bacteria are linked to inflammation and pathological conditions and there are abundant evidences showing that BEVs from pathogens are potential toxins and LPS carriers (see below); however, pathobionts are present at very low abundance in the gut and respiratory tract of healthy individuals in higher organisms for which the vast majority of BEVs identified in body samples are secreted by commensal bacteria (Cuesta et al., 2021). After gradient centrifugation of human stool samples from healthy control volunteers, the denser fractions (F8&9) were found to be significantly enriched in Firmicutes BEVs (89.6%). However, low-density BEVs exhibited a markedly elevated proportion of Proteobacteria and Bacteroidetes, exceeding the proportions observed in stool and F8&9 (Li et al., 2023).

Communication within and between different kingdoms is facilitated by EVs released by either the host cells or the gut microbiota, which can indirectly influence disease transmitting harmful signals (Díaz-Garrido et al, 2021). After uptake by eukaryotic cells, BEVs from bacterial pathogens will deliver pathogen-associated molecular patterns such as antigens or virulence factors.

Extensive research has substantiated the role of BEVs in the aetiology of various intestinal diseases, including inflammatory bowel disease (IBD) and colorectal cancer. Aggressive pathogens such as the highly virulent *E. coli* O104:H4 have been shown to secrete a cocktail of virulence factors via OMVs that bind and are actively transported into human intestinal epithelial cells (Caco-2) via dynamin-dependent endocytosis. Addition of purified OMVs (50 µg of protein) from this strain to Caco-2 cells delivers Shiga toxin 2a and Shigella enterotoxin 1, which induces caspase-9 and 3-mediated apoptosis, as well as H4 flagellin and O104 LPS, which induces interleukin-8 (IL-8) secretion from Caco-2 cells in culture (Kunsmann et al., 2015).

The presence of gut microbiota BEVs in the gut may be the cause of inflammation when the complement receptor of the immunoglobulin superfamily of macrophages (CRIg^+^) is reduced, for example, in the case of IBD. Along with decreased content of this receptor, increased content of BEVs in the mucosa of colonic tissue from IBD patients was found. Moreover, the highest content of bacterial DNA was found in the colon of mice CRIgKO with dextran sulphate sodium (DSS)-induced colitis after administration of 100 µg of BEVs/mouse. Microbial DNA within BEVs seems to be a main mediator in damaging the intestinal barrier function and triggering inflammation, which is mediated by the activation of the cGAS/STING pathway (Nie et al., 2024). Diet-induced obesity mice have more BEVs and a large proportion of them carried LPS, which entered the hepatic portal vein and reached the liver. In addition, some of the LPSs carrying BEVs escaped the liver and entered the peripheral blood circulation (Jain et al., 2024).

Previous research has substantiated the role of BEVs in the aetiology of various diseases, including IBD and colorectal cancer.

Primary sclerosing cholangitis (PSC) is frequently associated with IBD. In both preclinical model systems and in patients with PSC-IBD, the translocation of OMVs to the liver was found to correlate with enhanced bacterial sensing and accumulation of the NLRP3 inflammasome. The use of ductal organoids enabled the precise attribution of the pro-inflammatory and pro-fibrogenic properties of OMVs to signaling pathways dependent on Toll-like receptor 4 (TLR4) and NLRP3-gasdermin-D. The immunostimulatory potential of OMVs was confirmed in macrophages and hepatic stellate cells. Furthermore, the administration of gut pathobiont-derived OMVs to Mdr2^-/-^ mice resulted in a significant enhancement in liver inflammation and fibrosis. In a translational approach, the presence of OMVs in the systemic circulation and hepatic regions of severe fibrosis was substantiated using a PSC-IBD patient cohort. This showed that gut pathobionts released OMVs that cross the mucosal barrier, thus promoting liver inflammation and fibrosis in PSC-IBD (Dorner et al., 2024).

Fecal EVs isolated from patients with non-alcoholic steatohepatitis, but not those isolated from non-alcoholic fatty liver disease patients or from non-alcoholic fatty liver disease/non-alcoholic steatohepatitis donors, increased intestinal permeability, reducing expression of tight junction proteins and increased endothelial cell permeability and inflammatory cytokines and chemokines in the liver, which require the TLR4/LPS pathway. These authors demonstrate that these EVs actively participate in intestinal barrier dysfunction, followed by induction of liver inflammation and activation of hepatic stellate cells that start the liver fibrosis process (Fizanne et al., 2023).

### Role of bacterial extracellular vesicles in neurological and neurodegenerative diseases

Dysfunctions in gut microbiota (dysbiosis) are associated with chronic disease and mental disorders, brain dysfunction or cognitive impairment (Cuesta et al, 2021).

In samples from individuals with autism spectrum disorder (ASD), metagenomic analysis of bacterial DNA extracted from urinary BEVs revealed significantly altered microbiota profiles in individuals with ASD compared to controls. The bacterial patterns identified from urine BEVs were similar to those reported in faecal microbiota from ASD patients in the literature by different studies (Finegold et al., 2010; Adams et al., 2011; Kang et al., 2013; Strati et al., 2017). This study confirmed changes in the gut microbiota associated with ASD and suggests the potential use of urine BEVs to assess the microbiota in ASD (Lee et al., 2017).

An analysis of changes in the microbiota of patients with obsessive-compulsive disorder was performed by analyzing 16S ribosomal DNA extracted from BEVs isolated from the serum of controls and patients. Indeed, significant differences in α and β diversity and in microbiota composition of patients with this neurological disorder were found (Kang et al., 2023).

Wei et al. (2020) found that EVs isolated from the gut microbiota of patients with AD induced cognitive impairment and neuroinflammation when administered by intravenous injection every day for 8 weeks at a concentration of 50 µM to control mice. BEVs from healthy controls or patients did not show significant differences in concentration or size. A mean size of 100 nm was found in both BEV samples by nanoparticle tracking analysis and electron microscopy analysis. BEVs were able to cross the BBB, as they were detected in the hippocampus. Moreover, they damaged the BBB, increasing the Evans blue dye penetration and reducing the levels of the tight junction protein claudin-5. Injected mice showed impaired spatial memory and learning, assessed in the Water Morris Maze. BEVs also induced neuroinflammation in the hippocampus, increasing the expression of nuclear factor kappa B (NF-κB), tumor necrosis factor-α (TNF-α), and IL-1β, and increasing the numbers of microglia and astrocytes. Tau phosphorylation and glycogen synthase kinase‐3β activation play important roles in the pathogenesis of the disease and they were also affected by the injection of BEVs. These effects were not induced by BEVs from the gut microbiota of healthy controls (Wei et al., 2020). It has been reported a decrease of LPSs in the EVs isolated from blood of psychotic patients, compared with that isolated from controls, indicating decreased amount of BEVs in blood of these patients, likely due to dysregulated gut microbiota, and supporting a role of gut microbiota dysregulation in the neurological impairment in psychosis (Tunset et al., 2023). The utility of blood BEVs as biomarkers was also demonstrated in a mouse model of AD in which significant changes in the microbiota were detected by analyzing the metagenome included in these EVs (Park et al., 2017).

BEVs from *Akkermansia muciniphila* was administered to control mice by daily oral gavage of EVs at a concentration of 10 µg of protein in 200 µL for 4 weeks. The particle size of the isolated BEVs was variable, ranging from 30 to 150 nm. These BEVs increased serotonin levels in the hippocampus and altered gene expression of components of serotonin signaling and metabolism pathways to a greater extent than administration of the bacteria. However, the induction of neuroinflammation by these BEVs was not found in this study. BEVs may therefore be considered a mechanism by which the gut-brain-axis modulates serotonin signaling, contributing to the physiopathology of psychiatric disorders with altered serotonin neurotransmission (Yaghoubfar et al, 2020). Oral gavage daily for 5 days of BEVs (10 µg protein/kg) derived from *Paenalcaligenes hominis*, a bacterium which is more abundant in the gut of aged mice, induced cognitive impairment measured in the Y-Maze in control mice. The average BEV size was 0.43 ± 0.02 μm. Notably, effects induced by the BEVs were more potent than effects caused by orally administered LPSs. BEVs increased NF-κB^+^/Iba1^+^, LPS^+^/Iba1^+^, and IL-1R^+^ cell counts and reduced brain-derived neurotrophic factor (BDNF) expression in the hippocampus and increased IL-1β expression in the blood. FITC-conjugated BEVs and 16S RNA from bacteria were detected in the hippocampus. These effects on the hippocampus were all reduced by vagotomy, indicating that the vagus nerve contributes to the access of these BEVs into the brain (Lee et al., 2020). Recent observations by Ma et al. (2024) support the transport of bacterial EVs to the brain through the vagus nerve. They report that oral administration of EVs from feces of individuals with psychological stress and subclinical symptoms of depression or from *Escherichia fergusonii* cultures (an overpopulated bacteria found in these feces) induced depressive and anxiety-like behaviors and neuroinflammation in the hippocampus and cortex of control mice. The isolated BEVs were of a size range of 45–70 nm and 20 µg of EV protein was administered by oral gavage for 5 consecutive days to sham mice or mice with celiac vagotomy. These BEVs upregulated the expression of TNF-α, IL-1β, and IL-6 and the activation of NF-κB in the hippocampus, while reducing the expression of BDNF and IL-10. They also increased the expression of TNF-α, TLR-4, and NF-κB and reduced the expression of BDNF, serotonin, and its receptor 5HT1AR in the prefrontal cortex. Celiac vagotomy decreased the translocation of BEVs to the hippocampus and attenuated the pathologic effects on behavior and neuroinflammation (Ma et al, 2024).

*Helicobacter pylori* (HP) is one of the most prevalent pathogenic bacteria, which is present in the gastric epithelium of around 50% of the world population (Hooi et al, 2017). Moreover, chronic infection of HP has been correlated with several extra-gastric pathologies, including neurodegenerative disorders (Franceschi et al, 2015; Bravo et al, 2018; Doulberis et al, 2018). Park and Tsunoda (2022) proposed that BEVs produced in chronic HP infection in the gut induces neuroinflammation in the central nervous system, which would explain the higher prevalence of AD in people with HP infections. They found DNA from HP in the exosome fraction of sera from mice infected with HP, suggesting the presence of circulating BEVs from HP that could reach the brain. Results from *in vitro* experiments showed that BEVs (15 µg/mL) from HP increased cell proliferation, migration, and inflammatory cytokine production when added to microglial cultures. They also increased astrocyte whereas reduced neuroblastoma proliferation (Park and Tsunoda, 2022). Palacios et al. (2023) also studied the effects of BEVs from HP on brain cells. First, they demonstrate that fluorescently labeled BEVs reached the brain after intravenous injection in the tail of mice, and seemed to accumulate in the brain 24–72 hours post administration. BEVs induced astrocyte reactivity in the brain and neuronal damage, with less staining of neuronal bodies and loss and fragmentation of fibers. These results were supported by *in vitro* experiments, where BEVs from HP induced astrogliosis in primary astrocytes from neonatal rats, increasing the levels of connexin 43, glial fibrillary acidic protein, and vimentin. BEVs also increased the levels of molecules involved with astrocytes migration (syndecan-4, P2X7 receptor and the hemichannel pannexin 1) and the translocation of NF-κB to the nucleus, which resulted in higher interferon-γ and αVβ3 integrin production (Palacios et al., 2023). Another study by Xie et al. (2023) further explored the involvement of HP BEVs in AD. They demonstrate that HP BEVs loaded with Cre-recombinase and intragastrically administered to *Rosa26. tdTomato* reporter mice were able to cross various biological barriers and eventually reach the brain (with signal detected in the hippocampus and cortex), mainly co-localizing with astrocytes and to a lesser extent with neurons. The study found that HP BEVs promoted AD pathology in a mouse model of AD, inducing the formation of amyloid-beta (Aβ) plaques and microglia and astrocytes activation and reducing the number of microglia phagocytosing Aβ plaques. BEV administration induced memory loss, assessed in the Y Maze and Novel Object Recognition tests. Mechanistically, the C3-C3aR signaling pathway was identified as an important regulator in the detrimental effects on the brain mediated by HP BEVs. Blocking this pathway with pharmacological inhibition of C3aR prevented the dysfunction induced by HP BEVs on glial cells and neurons, reduced Aβ pathology, and improved cognitive function (Xie et al., 2023).

To date, there is no clear demonstration of the contribution of BEVs to the pathogenesis of Parkinson’s disease. However, as hypothesized by Koukoulis et al. (2024), BEVs released by gut bacteria are likely to trigger inflammation in the brain of patients and contribute to the progression of Parkinson’s disease.

These data are of clinical relevance, as reestablishing a healthy microbiome could be one strategy for reducing neuroinflammation and disease progression in patients with brain disorders.

### Oral infections and neurodegenerative disorders

In the last years, one of the aspects that has gained attention is the connection between oral health and neurodegenerative disorders. Periodontitis is an inflammatory disease mediated by bacteria that becomes prevalent in mid-life, probably at a similar age when the genesis of AD begins, but several years before the symptomatic manifestation of dementia (Liu et al., 2024). Periodontitis caused by *Porphyromonas gingivalis* (PG) is closely related to the development of AD. The presence of different molecules from PG has been detected in multiple regions of human brains and sera from AD patients, and the administration of PG or its virulence factors generates AD-like phenotypes in animal models, including neuroinflammation, cognitive impairments, or neurodegeneration (Liu et al., 2024). Singhrao et al. (2018) propose that PG BEVs could act as microbullets containing multiple virulence factors that can reach the brain due to its nanosized structure. PG BEVs may be recognized by the host’s pattern recognition receptors, inducing different transduction pathways that promote inflammation and microglial activation. Several studies point to the idea that a bacterial infection of the oral mucosa can promote neuroinflammation and cognitive impairment and that BEVs from these pathogenic bacteria would be involved in this process (Han et al., 2019; Ha et al., 2020, 2023; Gong et al., 2022; Nonaka et al., 2022; Pritchard et al., 2022; Ma et al., 2023).

*Aggregatibacter actinomycetemcomitans* is well known for causing periodontitis and systemic disease. When OMVs from this strain, with a size ranging 10–60 nm and containing small RNAs (miRNAs), are added to activated macrophages (cell line U937), the miRNAs are found in macrophages and induce increased production of TNF-α through activation of TLR-8. In addition, these authors showed that the intracardiac injection of DiD-labeled fluorescent BEVs containing miRNAs from this species in mice showed successful delivery to the brain after crossing the BBB 24 hours after injection, whereas at 4 hours, fluorescence was observed in brain capillaries only. These BEVs also increased the expression of TNF-α in the mouse cerebral cortex *in vivo* (Han et al., 2019). In a subsequent study, Ha et al. (2020) used intravital imaging analysis in monocyte-specific live CX3CR1-GFP mice intravenously injected through the tail veins with OMVs from this same bacterial strain. They observed that OMVs colocalized with meningeal macrophages 4–8 hours after injection and co-localization with microglial cells was only observed after 8 hours from the injection, suggesting that BEVs reach meningeal macrophages before microglial cells in the cortex. (Ha et al., 2020). In a posterior study, results obtained by Ha et al. (2023) support that BEVs from *A. actinomycetemcomitans* are involved in pathogenic pathways of neuroinflammatory diseases. The administration of BEVs (2.25×10^9^ particles (4.5 × 10^10^ particles/mL) Aa EVs in 50 µL of PBS) by intragingival injection or using a BEV-soaked gel strongly induces the production of proinflammatory cytokines TNF-α and IL-6 in the brain of mice. Moreover, these effects and the number of BEVs reaching the brain were more prominent when BEVs were administered to mice with periodontitis in comparison with control mice, indicating that the migration of BEVs from the oral cavity to the brain and their pathological effects in the brain would be facilitated when the physical barriers are compromised by periodontal diseases. Using TLR-reporter cell lines and MyD88 knockout mice, they confirmed that the release of these cytokines was triggered by BEVs from the bacteria via TLR4 and TLR8 signaling and their downstream MyD88 pathway, with concomitant activation of NF-κB signaling. These BEVs were also found to travel from axon terminals to the cell bodies of the trigeminal ganglion, where they increased the excitability of nociceptive neurons (Ha et al., 2023). This has implications not only in dementia-like behaviors, but also in neuropathic pain or neuralgia.

Gong et al. (2022) found that oral gavage administration of BEVs (4 mg/kg every other day for 8 weeks) from PG strain ATCC 33277 using feeding needles induced memory dysfunction in mice, impairing learning, spatial reference memory, and memory consolidation. The authors found that these BEVs, with an average diameter of 115 nm and labeled with DiO, reached the brain and increased tau phosphorylation in the hippocampus, which is closely related to memory consolidation and one of the hallmarks of AD pathology. The BEVs also increased the number of microglia and astrocytes in the hippocampus, as well as the number of cells positive for IL-1β. NLRP3 inflammasome activation is involved in this process, driving microglia activation and tau phosphorylation. Results from *in vitro* experiments showed increased extracellular ASC (apoptosis-associated speck-like protein) and NLPR3 expression in microglial BV2 cells after stimulation with BEVs from PG. Moreover, N2a neurons treated with conditioned medium from BV2 cells stimulated with the BEVs, showed higher levels of phosphorylated tau (Gong et al., 2022). In a similar study, already described above, gingival exposure, but not oral gavage, to BEVs from PG increased TNF-α and IL-1β and reduced IL-10 and BDNF in the hippocampus of mice. These BEVs induced microglial activation, promoting LPS^+^Iba1^+^, NF-κB^+^Iba1^+^, and TLR4^+^Iba1^+^ cell populations in the hippocampus, which must be the source of the altered cytokines. The induction of neuroinflammation leads to impaired memory. The mechanism involves translocation of BEVs into the brain through the trigeminal nerve, as they have been found in the trigeminal ganglia (Ma et al., 2023). Effects of PG BEVs on neuroinflammation have also been studied in the zebrafish model by Adewoyin et al. (2024). In this study, a single injection of BEVs induced an increase in NO levels, IL-1β and IL-6 and PGE2 production in the brain at different time points after injection (between 45 minutes and up to 24 hours), accompanied by the beginning of glial cells activation, astrocytosis, signs of edema and necrosis (Adewoyin et al., 2024).

These studies indicate that BEVs from oral pathogens can be a risk factor for dementia and cognitive decline, and even be a triggering factor to initiate AD pathology. This is of special relevance because this is a modifiable risk factor; treatment of oral infection could help to prevent the onset or development of AD.

### Action mechanisms of pathogenic bacterial extracellular vesicles

#### Blood–brain barrier disruption

A main mechanism by which BEVs can cross the BBB is by increasing its permeability. This has been reported for BEVs from some pathogenic bacteria, and is mediated by reduction (often by cleavage or proteolysis) of Tight junctions and other membrane proteins of brain endothelial cells involved in BBB formation and of extracellular matrix proteins. Circulating BEVs could be absorbed by brain endothelial cells, disrupting cellular and subcellular structures and functions, leading to cell death and leaks in the endothelial barrier (Liu et al., 2024).

BEVs from PG labeled with the fluorescent tracer DiO were detected in the hippocampus and cortex of mice 3 days after oral administration. It was observed that these BEVs increased BBB permeability after 8 weeks of oral gavage, reducing the gene expression of the tight junction proteins claudin-5, ZO-1, and occludin in the hippocampus (Gong et al., 2022).

Farrugia et al. (2020) found that BEVs from PG increased vascular permeability *in vitro* by cleavage of PECAM-1, a major endothelial adhesion protein, which is found at BBB adherent junctions. BEVs from PG containing gingipains could be weakening the integrity of tight junctions of the human BBB, as they reduced the electrical resistance and recovery deficits, as well as the content of ZO-1 protein, when applied to a monoculture of human brain microvascular endothelial cells (Pritchard et al., 2022). Nonaka et al. (2022) highlighted the role of gingipains in the disruption of the BBB by PG BEVs, which reduced the levels of ZO-1 and occludin in hCMEC/D3 (human cerebral microvascular endothelial cell line) cell monolayer. BEVs isolated from feces of AD patients, but not those isolated from controls, disrupted the BBB, increasing the Evans blue dye penetration and reducing the levels of the tight junction protein claudin-5 (Wei et al. 2020).

It should be noted that increased BBB permeability not only allows access to the brain of BEV contents, which may induce brain damage, but also facilitates that other neurotoxic agents may access the brain through the BBB disrupted by BEVs, thus causing additional pathological effects.

In other studies, BEVs have also been shown to cross the BBB, but without inducing alterations in its permeability, as shown by Xie et al. (2023).

Mi et al. (2023) showed that BEVs from Salmonella do not cross the BBB in mice with glioma, which makes the BBB more impermeable to drugs. However, the bacteria themselves disrupt the BBB, allowing access to BEVs that are endocytosed by neutrophils through the TLR4 receptor present on their membrane, which recognizes pathogen-associated molecular patterns on the BEV membrane. In this review, it was demonstrated that these BEVs efficiently increase the penetration of an antitumor drug (doxorubicin) into glioma. The drug alone does not reach the brain in spite that the previous inoculation of bacteria has increased the BBB permeability. BEVs loaded with the drug facilitate its access to neutrophils that invade the tumor, introducing the drug into the tumor efficiently.

Yu and Kim (2012) analyzed the mechanism involved in the invasion of human endothelial cells by a meningitis inducing *E. coli* strain. They showed that incorporation of the cytotoxic necrotizing factor 1 bacterial toxin in OMVs and its posterior delivery into endothelial cells is necessary for invasion of endothelial cells by this strain of *E. coli.*

#### Transference of RNAs and microRNAs to the brain

Choi et al. (2017) showed that OMVs from periodontal pathogens labeled with DiO and containing miRNAs penetrated cells after incubation with a fibroblast cell line. Han et al. (2019) observed that BEVs from *Aggregatibacter actinomycetemcomitans* carry RNAs that can be delivered to human macrophage cells, where they modulated the expression of TNF-α, TLR-8, and NF-κB phosphorylation. In a subsequent study, Ha et al. (2020) found that BEVs from these same bacteria can successfully deliver RNAs into brain monocyte/microglial cells and cause neuroinflammation in mice. Using intravital imaging analysis, they demonstrate that BEVs are transported to brain microglial cells after intracardiac injection and through the meninges. Microglial cells internalized RNAs carried by the BEVs, which activated NF-κB and the production of IL-6 in BV2 cells (Ha et al., 2020).

Other mechanisms of bacterial pathogenicity involve EVs, although not derived from the bacteria themselves, but induced by them. Rickettsia are pathogenic primarily by targeting and altering endothelial cell function, and EVs derived from infected endothelial cells are enriched in miRNA23a, a negative regulator of ZO-1 mRNA. EVs from these Rickettsia-infected cells alter human brain endothelial cell function through the release of miRNA23a, which decreases ZO-1 mRNA (Zhou et al., 2022). This is also likely a mechanism of BBB disruption mediated by EVs due to infection with pathogenic bacteria.

#### Activation of macrophages/microglial cells

One possibility is that circulating immune cells, especially macrophages, phagocytose the BEVs and transport them or their components into the brain like a “Trojan horse” (Liu et al., 2024).

Several components and virulence factors carried by the BEVs can directly activate microglia and promote neuroinflammation when the BEVs reach the brain. LPS/lipoprotein from PG is known to stimulate pattern recognition receptors on innate immune cells to activate immune responses and is a major component of the PG BEVs.

The work of Ma et al. (2023) also shows the induction of activated microglia by BEVs from PG after gingival exposure. An increase in double-positive cells for Iba1 (microglia) and LPS, gingipain, or NF-κB, a transcription factor that regulates cytokine synthesis, and an increase in pro-inflammatory cytokines and a reduction in anti-inflammatory ones were detected in the mouse hippocampus. Moreover, oral gavage of BEVs from *Paenalcaligenes hominis* induced microglia activation (increase in Iba1^+^/NF-κB^+^ cells) in the mouse hippocampus, along with memory impairment (Lee et al., 2020).

A summary of the pathological effects of BEVs is shown in **Additional Table 1**.

**Additional Table 1:** Pathological and beneficial effects of BEVs

Bacterial specie	Host model	Effect	Reference
**SYSTEMIC EFFECTS**			
***Pathological effects***			
*Escherichia coli* O104:H4	Human intestinal epithelial cells	Apoptosis and IL-8 secretion	Kunsmann et al., 2015
---------------------	DSS mice model of IBD	Gut inflammation	Nie et al., 2024
Gut pathobiont	PSC-IBD	Liver inflammation and fibrosis	Dorner et al., 2024
Fecal BEV non-alcoholic steatohepatitis	Intestinal and endothelial cells	Increased intestinal barrier permeability and inflammation	Fizanne et al., 2023
***Beneficial effects***			
*Bacteroides fragilis* BEV	Dendritic cells IBD Mice model	Enhance regulatory T cells and anti-inflammatory cytokine production Improves colitis	Shen et al., 2012
*Lactobacillus rhamnosus* GG and *Lactobacillus reuteri* DSM 17938 BEV	T^-^ and NK cells	Reduce induced interferon-γ and IL-17A responses	Mata Forsberg et al., 2019
*Lacticaseibacillus rhamnosus* JB-1	Dendritic cells Intestinal epithelial human cell line	Activation of TLR-2 and an increase in IL-10 Reduction of IL-8 after TNF-α stimulation	Champagne-Jorgense n et al., 2021a
*Lactobacillus rhamnosus* GG	IBD Mice model	Inhibition of TLR4-NF-κB-NLRP3 inflammasome axis activation Improvement of intestinal inflammation Reduction of colon tissue damage	Tong et al., 2021
*Lactobacillus paracasei* BEV	LPS-induced inflammation of HT29 intestinal cells	Increases anti-inflammatory proteins and reduces those proinflammatory	Choi et al., 2020
*Limosilactobacillus reuteri* BBC3 BEV	Chicken jejunum	Increases anti-inflammatory genes and reduces those proinflammatory	Huet al., 2021
*Limosilactobacillus. reuteri* strains DSM17938 and BG-R46 BEV	Rat dorsal root ganglion cells	Antagonize TRPV1	Pang et al., 2022
*Lactobacillus rhamnosus* BEV	Liver cancer cells HepG2	Increase of the apoptotic index (bax/bcl2 expression ratio)	Behzadi et al., 2017
*Bacteroides thetaiotamicron* BEV	IBD mouse model, bone marrow-derived monocytes	Increase in IL-10 and improve survival	Fonseca et al., 2022
*Roseburia intestinalis* BEV	IBD mouse model	Reduce inflammation	Han et al., 2024
*Bifidobacterium longum* KACC 91563 BEV	Mice	Alleviate food allergy and induce apoptosis in mast cells	Kim et al., 2016
*Bifidobacterium longum* BEV	Co-cultures of splenocytes and dendritic-CD4+T cells	Anti-inflammatory, induce IL-10 secretion	Mandelbaum et al., 2023
*Bifidobacterium longum* BEV	Mouse model ofhepatocelular carcinoma, liver	Reduce liver fibrosis, apoptosis, tumor formation rate, and improve liver function, down-regulate transforming growth factor-β1 expression and Smad3 phosphorylation	Li et al., 2024b
Lactobacillus, Bifidobacterium, and Lactococcus BEV	Partial hepatectomy in mice Liver sinusoidal endothelial cells	Enhance neutrophil clearance, reduce intrahepatic VCAM1 and ICAM2 expression, improve liver regeneration, reduce IL17 expression	Cohen et al., 2023
**BRAIN EFFECTS**			
***Pathological effects***			
Urine BEV ASD			Lee et al., 2017
Gut BEV AD Patients	Mice Hippocampus	BBB Disruption Neuroinflammation Cognitive impairment	Wei et al., 2020
BEV Psychotic patients	Blood	*Decreased content*	Tunsetetal.,2023
*Akkermansia muciniphila* BEV	Mice Colon Hippocampus and serum	Increased serotonin and signaling proteins in the colon and hippocampus, and decreased in serum	Yaghoubfar et al., 2020
*Paenalcaligenes hominis* BEV	Control mice Hippocampus and blood	Microglia activation, Neuroinflammation, Dementia-like behavior	Lee et al., 2020
*Escherichia fergusonii* BEV	Control mice Hippocampus and Cortex	Neuroinflammation, Anxiety and depression, Serotonin	Ma et al., 2024
*Helicobacter pylori* BEV	Microglia cultures	Proliferation, migration, increase in proinflammatory cytokines	Park and Tsunoda, 2022
*Helicobacter pylori* BEV	Mice and astrocyte culture	Astrocyte reactivity and neuronal damage	Palacios et al., 2023
*Helicobacter pylori* BEV	Mice model of AD	Formation of Aβ plaques and microglia and astrocytes activation, memory impairment	Xie et al., 2023
*Aggregatibacter actinomycetemcomitans* BEV	Mouse brain	Increase of TNF-α expression	Han et al., 2019
*Aggregatibacter actinomycetemcomitans* BEV	Mouse brain periodontitis	Production of TNF-α and IL-6. Activation of TLR4, MyD88 and NF-κB Increase the excitability of nociceptive neurons	Ha et al., 2023
*Porphyromonas gingivalis* BEV	Brain AD	Inflammation, glial activation, accumulation of Aβ and NFTs	Singhrao et al., 2018
*Porphyromonas gingivalis* BEV	Mouse hippocampus and microglia culture	Memory and learning impairment. Increased Tau phosphorilation, astrocytes and microglia and IL-1β, NLRP3 inflammasome activation	Gong et al., 2022
*Porphyromonas gingivalis* BEV	Zebrafish	Neuroinflammation, astrocytosis, edema, necrosis	Adewoyin et al., 2024
*Porphyromonas gingivalis* BEV	Monoculture of human brain microvascular endothelial cells	Increase vascular permeability, cleavage of PECAM-1 Decrease electrical resistance and ZO-1 levels	Farrugia et al., 2020 Pritchard et al., 2022 Nonaka et al., 2022
*Aggregatibacter actinomycetemcomitans* BEV	Microglia culture	Internalization of exRNAs	Ha et al., 2020
***Beneficial effects***			
*Lactobacillus plantarum* BEV	Cultured hippocampal neurons and mice	Increase the expression of neurotrophic factors, antidepressant-like effects	Choi et al., 2019
*Lactobacillus plantarum* BEV	Ischemic brain tMCAO mice and OGD/R-induced neurons	Inhibit neuronal apoptosis	Yang et al., 2022
*Lactobacillus rhamnosus* (ATCC 7469) BEV	Microglia cell culture	Reduce the proinflammatory (M1) markers and increase expression of anti-inflammatory markers of M2	Yang et al., 2024
*Lactobacillus paracasei*	Mouse hippocampus	Reversion of stress-induced depressive behavior	Kwon et al., 2023a
BEV			
*Lactobacillus paracasei* BEV	APP/PS1 transgenic mouse model of AD	Reduction of neuroinflammation and cognitive impairment	Kwon et al., 2023b
*Lactobacillus paracasei* BL23 BEV	Hyperammonemic rats, model of hepatic encephalopathy. Cerebellar ex vivo slices of hyperammonemic rats	Improve motor function, reduce cerebellar neuroinflammation, and restore altered GABAergic neurotransmission	Arenas et al., 2024
*Lactobacillus paracasei* BL23 BEV	Hyperammonemic rats, model of hepatic encephalopathy. Hippocampal ex vivo slices of hyperammonemic rats	Improve cognitive function, reduce hippocampal neuroinflammation, and restore altered glutamatergic neurotransmission	Izquierdo-Altarejos et al., 2025

AD: Alzheimer's disease; APP: amyloid precursor protein: ASD: autism spectrum disorder; BBB: blood-brain barrier; BEV: bacteria extracellular vesicles; DSS: dextran sulphate sodium; IBD: inflammatory bowel disease; ICAM: intercellular adhesion molecule; IL: interleukin; LPS: lipopolysaccharide; NFkB: nuclear factor kappa B; NFTs: neurofibrillary tangles; NK: natural killer; NLRP: NOD-like receptor Pyrin domain containing; OGD/R: oxygen glucose deprivation/re-oxygenation; PECAM-1: platelet/endothelial cell adhesion molecule 1; PS1: preseniline-1; PSC: primary sclerosing cholangitis; TLR: Toll-like receptor; tMCAO: transient middle cerebral artery occlusion; TNF: tumor necrosis factor; TRPV1 : transient receptor potential cation channel subfamily V member 1 (vanilloid receptor 1); VCAM: vascular cell adhesion protein 1.

## Beneficial Effects of Bacterial Extracellular Vesicles in Hosts

The beneficial use of probiotic bacteria in health improvement and their therapeutic use is widely reported. Secretion of BEVs has recently been linked to a possible mechanism of action (Shen et al., 2012; Pirolli et al., 2021; Gilmore et al., 2021; Li et al., 2024b). Shen et al. (2012) showed that OMVs from the human commensal *Bacteroides fragilis* target TLR2 in dendritic cells, leading to enhanced regulatory T cells and anti-inflammatory cytokine production. Oral feeding of these OMVs improves colitis in a preclinical model consisting of 2,4,6-trinitrobenzene sulfonic acid-induced intestinal inflammation in mice.

Gram-positive-derived BEVs are a better option as these BEVs lack toxic LPSs and other pathologic mediators. The therapeutic use of BEVs is mainly focused on its immunomodulatory effect. In addition, one of the main advantages of using BEVs for the therapeutic approach in several pathologies is that they are more easily modified through bioengineering, so that their cargo, activity, location, and targeting, and even their functions, can be modified, to improve their therapeutic potential. The expression and further encapsulation of bioactive molecules into natural nanoparticles produced by probiotic bacteria could have practical implications in food, nutraceuticals, and clinical therapies (Gilmore et al., 2021; Palomino et al., 2021; Doré and Boilard, 2023; Taitz et al., 2023; Li et al., 2024a).

Different positive effects of BEVs from commensals and probiotics have been reported, mainly of BEVs from Lactobacillus, and mainly by modulating the immune system and in different pathological conditions and diseases. Cell-free supernatants of *Lactobacillus rhamnosus* GG and *Lactobacillus reuteri* DSM 17938 effectively reduced interferon-γ- and IL-17A-induced responses in T- and NK cells. Protein analysis highlighted the presence of Lactobacillus-induced IL-1 receptor antagonist as the candidate responsible for this activity. This effect is due to soluble factors, possibly BEVs (Mata Forsberg et al., 2019). When the strain *Lacticaseibacillus rhamnosus* JB-1 was administered to mice, BEVs of this strain could be isolated from blood within 2.5 hours. Administration of *L. rhamnosus* JB-1 BEVs had the same effect on the immune system as purified lipoteichoic acid from the original bacteria. They activated TLR2 and increased IL-10 expression by dendritic cells and reduced IL-8 after TNF-α stimulation in an intestinal epithelial cell line. This study supports that oral consumption of live bacteria rapidly leads to the circulation of their BEVs, suggesting that there is a nanoparticle pathway by which probiotics and other beneficial bacteria may systemically act on the hosts (Champagne-Jorgensen et al., 2021b).

The beneficial effect of BEVs has been mainly studied in intestinal inflammation diseases, colitis, and IBD (Zubair et al., 2024). In mice with DSS-induced colitis, BEVs from *L. rhamnosus* (GG strain) had a powerful anti-inflammatory effect, reducing pro-inflammatory cytokines (TNF-α, IL-1β, IL-6, and IL-2) and ameliorating intestinal inflammation by inhibiting TLR4-NF-κB-NLRP3 axis activation. These BEVs prevented colonic tissue damage and restored the gut microbiota homeostasis (Tong et al., 2021). BEVs from a closely related species, *L. paracasei*, reduced the expression of the pro-inflammatory cytokines IL-1α, IL-1β, IL-2, and TNF-α, decreased the activation of inflammation-associated proteins, such as cyclooxygenase-2, inducible nitric oxide synthase, and NF-κB, and increased the expression of the anti-inflammatory cytokines IL-10 and transforming growth factor-β (TGF-β), in LPS-induced inflammation of HT29 intestinal cells (Choi et al., 2020). *Limosilactobacillus* (formerly Lactobacillus) *reuteri* BBC3-derived BEVs suppressed the LPS-induced expression of pro-inflammatory genes and improved the expression of anti-inflammatory genes (IL-10 and TGF-β) in the jejunum, and they could be internalized by chicken macrophages. *L. reuteri* BEVs carried several bioactive components, such as RNA, DNA, and proteins such as glucosyltransferase, serine protease, and elongation factor Tu, which have been proposed to mediate this anti-inflammatory effect (Hu et al., 2021). *L. reuteri* strain DSM17938 is a probiotic with abundant scientific evidence for the attenuation of the infantile colic condition, where transient receptor potential vanilloid 1 (TRPV1) could be involved in pain perception. Interestingly, BEVs from *L. reuteri* strains DSM17938 and BG-R46 could antagonize TRPV1 in a rat dorsal root ganglion cell model (Pang et al., 2022).

The possible use of BEVs for cancer treatment has also been evaluated. For example, BEVs from *Lactobacillus rhamnosus* increased the apoptotic index (bax/bcl2 expression ratio) in the liver cancer cells HepG2 (Behzadi et al., 2017).

BEVs from other beneficial bacteria also shows beneficial, mainly anti-inflammatory, effects. *Bacteroides thetaiotamicron* (Bt) is one of the new generation of probiotics. In the DSS-induced colitis model in mice, Bt BEVs increased anti-inflammatory IL-10 production, improving survival. Most remarkably, histone H3K4me1 methylation analysis in bone marrow-derived monocytes showed that Bt-EVs induced epigenetic reprogramming that persisted after LPS challenge (Fonseca et al., 2022). Bacteria from the Lachnospiraceae family have been implicated in the formation of butyrate and other beneficial short-chain fatty acids in the gut. A prominent genus in this family is Roseburia, and within this genus, *R. intestinalis* has been proposed to have beneficial effects on the organism. Using the mouse model of DSS-induced colitis, *R. intestinalis*-derived BEVs were shown to be potent anti-inflammatory mediators. Orally administered BEVs accumulated in inflamed colonic tissue and promoted intestinal proliferation of Bifidobacterium and reduced inflammation (Han et al., 2024).

Bacteria of the bifidus group exert anti-inflammatory effects. *Bifidobacterium longum* KACC 91563 BEVs alleviated food allergy in mice by binding specifically to mast cells and inducing apoptosis without affecting T-cell immune responses (Kim et al., 2016). *B. longum* BEVs induced IL-10 secretion from co-cultures of splenocytes and dendritic-CD4^+^ T cells. These BEVs were enriched in ABC transporters, quorum sensing proteins, and extracellular solute-binding proteins, for which a prominent function in the anti-inflammatory profile of strains of *B. longum* had been described (Mandelbaum et al., 2023). *B. longum* BEV administration reduced liver fibrosis, apoptosis, and oxidative stress induced by diethylnitrosamine in a mouse model of hepatocellular carcinoma. This work demonstrated that these BEVs could enter hepatocytes. Administration of these BEVs reduced the tumor formation rate and improved liver function, down-regulating TGF-β1 expression and Smad3 phosphorylation in mouse liver (Li et al., 2024a). BEVs from *Lactobacillus*, *Bifidobacterium*, and *Lactococcus* translocated to the liver after surgery, reducing intrahepatic VCAM1 and ICAM2 expression and improving liver regeneration at 72 hours, with a significant reduction in IL-17 expression (Cohen et al., 2023). Unfortunately, the strains and species were not properly described.

BEVs from different species of non-classical probiotic strains of lactic acid bacteria had different, pro- or anti-inflammatory effects, depending on the cellular activation state. For example, BEVs from *L. plantarum*, *Leuconostoc mesenteroides*, and *Latilactobacillus curvatus* increased the production of nitric oxide, TNF-α, and IL-6 in macrophages (RAW264.7) and mice. However, paradoxically, these BEVs prevented inflammation in the LPS-stimulated microglial cells, possibly by inhibiting the extracellular signal-regulated kinase and p38 signaling pathways (Kim et al., 2022). Similarly, BEVs from *L. reuteri* upregulated IL-1β and IL-6 from peripheral blood mononuclear cells if they were not previously stimulated, but they reduced interferon-γ and TNF-α responses when these cells were challenged with *Staphylococcus aureus*, and they also restored affected epithelial integrity in transwell cultures of Caco-2/HT29-MTX epithelial cells (Pang et al., 2022).

### Beneficial effects on brain function and neurological/neurodegenerative diseases

Considering the importance of the microbiota-gut-brain axis in mental health, it is likely that BEVs could also induce beneficial effects in brain diseases (Haas-Neill and Forsythe, 2020; Pirolli et al., 2021; Louka and Koumandou, 2024; Mottawea et al., 2025). There are yet few studies reporting beneficial effects of BEVs on the brain and neurological and neurodegenerative diseases. Beneficial effects of BEVs have been reported for AD, stress and anxiety-depression symptoms, ischemic brain injury, and in hyperammonemia and hepatic encephalopathy.

**Figure 3:**
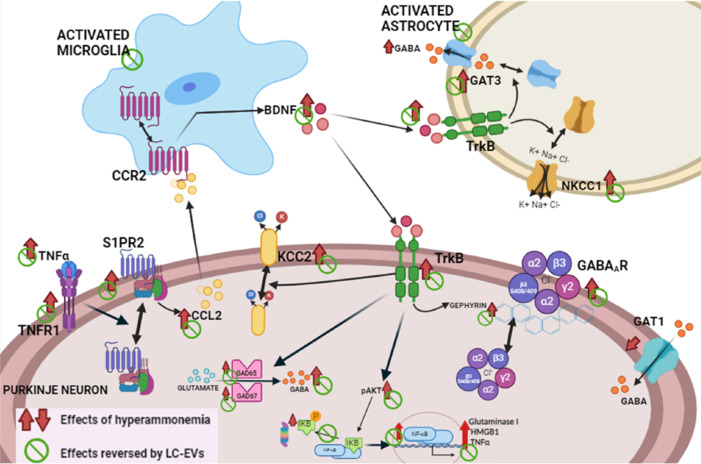
Extracellular vesicles from *L. paracasei* (LC-EV) reverse the activation of the pathways by which neuroinflammation enhances GABAergic neurotransmission in the cerebellum and motor incoordination in hyperammonemic rats. Hyperammonemia-induced neuroinflammation activates the TNFR1-S1PR2-CCL2-CCR2 pathway. Increased BDNF enhances activation of TrkB in Purkinje neurons, leading to increased levels of the GABA synthesizing enzymes and GABA. Enhanced TrkB activation also increases the membrane expression of GABAA receptor subunits and KCC2. Moreover, enhanced activation of TrkB in activated astrocytes increases membrane expression of GAT3 and of NKCC1, which also contributes to enhancing extracellular GABA. This results in enhanced GABAergic neurotransmission and motor incoordination. All these changes are reversed by treatment with LC-EVs. The effects of hyperammonemia are indicated by red arrows (↑) and the effects reversed by LC-EVs by green symbol ᴓ. Reprinted with permission from Arenas et al. (2025). CCL2: C-C motif chemokine ligand 2; CCR2: CC motif chemokine receptor 2; GABA: γ-aminobutyric acid; GAT3: GABA transporter 3; KCC2: potassium chloride cotransporter 2; LC-EV: *Lactobacillus paracasei* *BL23* extracellular vesicles; NKCC1: sodium-(potassium)-chloride cotransporter 1; S1PR2: sphingosine-1-phosphate receptor 2; TNFR1: tumor necrosis factor receptor 1; TrkB: tropomyosin receptor kinase B.

Choi et al. (2019) showed that *Lactobacillus plantarum*-derived BEVs increase the expression of the neurotrophic factor BDNF when added at a concentration of 20 µg/mL to a hippocampal cell line (HT22) treated with glucocorticoids that have decreased BDNF content. This effect is mediated by Sirt1, which modulates BDNF expression. In addition, intraperitoneal injection (0.1 to 0.27 µg/kg) of these BEVs to mice with stress-induced depressive-like behavior reduced this behavior, both during the stress phase and later. These BEVs increased the content of BDNF in the hippocampus of mice with stress-induced depressive-like behavior. Then, BEVs from this probiotic were suggested as a good antidepressant treatment (Choi et al., 2019). BEVs from this species inhibit neuronal apoptosis both in the *in vitro* and *in vivo* mouse models of ischemic injury. These BEVs, with an average diameter of 67 nm, reach the brain. Apoptosis is mediated by cFos in these models, and BEVs reduced cFos through increased expression of the miR-101a-3p, which directly targets cFos that activate TGF-β1 to induce apoptosis. In addition, downregulated Hsa-miR-101–3p was found in the plasma of patients with ischemic stroke, but it was upregulated in the patients with neurological recovery after intravenous thrombolysis (Yang et al., 2022). The effect of BEVs from *Lactobacillus rhamnosus* (ATCC 7469) on polarization of microglial cells *in vitro* was analyzed by Yang et al. (2024), who reported that these BEVs added *in vitro* were internalized by microglial cells and reduced the proinflammatory (M1) markers induced by LPSs, whereas increasing the expression of anti-inflammatory markers of M2 microglia. The ability of BEVs from *Lactobacillus paracasei* to affect wide genome changes in the model of chronic stress was also studied. These BEVs (10 µg/mL) counteract some gene expression changes, assessed with microarray analysis, induced by corticosterone in the HT22 cells, restoring mainly the altered MAPK signaling pathway. These changes were also reversed in the hippocampus of mice with stress-induced depressive-like behavior by these BEVs, administered at 6 µg/kg, effectively improving stress-induced depressive-like behavior (Kwon et al, 2023a). A similar study was carried out in models of AD. *L. paracasei* BEVs (10 µg/mL) also modify β-amyloid-induced transcriptional changes in HT22 cells, restoring the reduced levels of BDNF, neurotrophins, and matrix metalloproteinases 2 and 9, through upregulation of MeCP2 and Sirt1. In the animal model of AD, Tg-APP/PS1 mice, these BEVs also induced upregulation of these factors, an increase in the neurotrophic factors, and reduced amyloid-β accumulation and neuroinflammation in the hippocampus, improving the cognitive impairment. In this case, *in vivo* treatment with the BEVs was performed by oral administration at a dose of 2.27 mg/kg/d in the drinking water for 1.5 months. BEVs were diluted in drinking water to 15 µg/mL (1.29 × 10^9^ EV particles/mL) (Kwon et al., 2023b). Arenas et al. (2024) showed that intravenous administration of 50 μg of protein (equivalent to 2 × 10^12^ particles) per rat of BEVs from *L. paracasei* BL23 (LC-EVs), once per week for 4 weeks, to rats with chronic hyperammonemia and minimal hepatic encephalopathy improves motor coordination. The average size of these BEVs was 19 nm. In the cerebellum of hyperammonemic rats, glial activation and neuroinflammation increase TNF-α levels that trigger the activation of the TNFR1-S1PR2-BDNF-TrkB and the TNFR1-TrkB-pAKT-NF-κB-glutaminase-GAT3 pathways, leading to enhanced GABAergic neurotransmission, which induces motor incoordination. Arenas et al. (2024) showed that intravenous injection of LC-EVs reverses glial activation and neuroinflammation in the cerebellum *in vivo* in hyperammonemic rats and restores motor coordination and locomotor gait alterations. Moreover, *ex vivo* treatment (10 μg of protein, equivalent to 4 × 10^11^ EVs) of cerebellar slices from hyperammonemic rats with LC-EVs also reverses glial activation and neuroinflammation and normalizes the activation of the TNFR1-S1PR2-BDNF-TrkB and the TNFR1-TrkB-pAKT-NF-κB-glutaminase-GAT3 pathways and GABAergic neurotransmission (**Figure 3**). These data support that the beneficial effects of LC-EVs are produced directly in the cerebellum. This is also one of the first evidence that BEVs can modulate neurotransmission and intracellular signaling in the brain. The results reported here suggest that LC-EVs may be a useful therapeutic tool to improve motor coordination and locomotor gait in cirrhotic patients with hepatic encephalopathy.

Using the same animal model of hyperammonemia and minimal hepatic encephalopathy, and the same treatment pattern, Izquierdo-Altarejos et al. (2025) analyzed the effects of LC-EVs on cognitive function and the underlying mechanisms. They showed that intravenous injection of LC-EVs reverses glial activation in the hippocampus and restores cognitive function in hyperammonemic rats as assessed in the radial maze. *Ex vivo* studies in hippocampal slices show that hyperammonemia increases TNF-α and TNFR1 and S1PR2 membrane expression and activation, leading to increased IL-1β content and activation of IL-1 receptor and Src. This increases CCL2 and BDNF, and TrkB activation. This leads to increased membrane expression of the NR2B subunit of the NMDA receptor and the GluA2 subunit of AMPA receptors and reduced membrane expression of the GluA1 subunit. This would be responsible for cognitive impairment. EVs from *L. paracasei* reduce neuroinflammation in hyperammonemic rats and restore the function of the TNF-α-TNFR1-S1PR2-IL-1β-CCL2-BDNF-TrkB pathway, glutamatergic neurotransmission, and cognitive function in rats with hyperammonemia and MHE. This suggests that these EVs could also improve cognitive function in cirrhotic patients with MHE (Izquierdo-Altarejos et al, 2025).

A summary of the beneficial effects of BEVs is shown in **Additional Table 1**.

### Engineered bacterial extracellular vesicles

Different strategies have been developed to improve the therapeutic capacity of BEVs. For example, *L. plantarum* and *L. casei* were cultured under different conditions to increase the anti-inflammatory potential of their BEVs in macrophage inflammation models. The IL-10 content was increased and the TNF-α content was reduced in the BEVs of these bacteria, thus increasing their anti-inflammatory efficacy (Müller et al., 2021).

There is also a current trend to improve the therapeutic use of BEVs by transforming or modifying their characteristics through bioengineering. One of the main applications of these techniques is to directly load BEVs with drugs to deliver them more safely and effectively to their specific targets.

Modifications through bioengineering techniques expand the therapeutic potential of EVs in general, and also of BEVs. Several studies have addressed this possibility through different strategies to improve different pathological situations.

One possibility is to load BEVs with beneficial molecules to be delivered in the host after administration of the BEVs, acting as drug delivery systems (Liu et al., 2021). OMVs have been used to engineer non-pathogenic *E. coli* to deliver small interfering RNA targeting kinesin spindle protein to kill human cancer cells *in vitro* and by xenotransplantation into mice (Gujrati et al., 2014). OMVs loaded with melanin from *E. coli* expressing human tyrosinase have also been used in optoacoustic tomography and for light (heat) inactivation of subcutaneous 4T1 mouse mammary gland tumors (Gujrati et al., 2019).

Drug delivery through BEVs is particularly interesting for brain pathologies as they are a good vehicle to cross the BBB and BEVs are easy to obtain and to modify for brain targeting (Chen et al., 2022). Chen et al. (2022) used the BBB invasion ability of the *E. coli* K1 (EC‐K1) outer membrane to construct a biomimetic self‐assembled nanoparticle with a surface featuring a lipopolysaccharide‐free EC‐K1 outer membrane for brain‐targeted drug delivery. These biomimetic nanoparticles crossed the BBB through the transcellular vesicle transport pathway. These authors successfully demonstrated that the modified nanoparticles can circulate intracranially through the interstices for a long time, can transport drugs, and release them inside the brain with high biocompatibility.

Another approach using OMVs was to generate complexes between gold nanoparticles and *E. coli* OMVs to treat glioblastoma in mice. Tumor growth was slowed with this complex because it increases reactive oxygen species that attack cancer cells, increasing the effect of radiotherapy (Chen et al., 2021). Another study was conducted in a model of cerebral ischemia, based on introducing the drug into bacterial OMVs, so that these OMVs are incorporated into neutrophils after activating their membrane receptor TLR2. The neutrophils reach the ischemic area of the brain after chemoattraction from the damaged zone and release the OMVs containing the drug, which is then released, thus increasing the presence of the drug in the ischemic area and therefore increasing its efficacy (Pan et al., 2023). Tumor migration of neutrophils was also used for the delivery of doxorubicin-loaded OMVs. The surface pathogen-associated molecular patterns derived from native bacteria allowed that OMVs/doxorubicin could be selectively recognized by neutrophils, thus facilitating glioma-targeted delivery of the drug (Mi et al., 2023).

The studies summarized in this review show that BEVs play a key role in the mechanisms of communication between the microbiota and the host. BEVs are involved in the pathophysiology of many diseases, including neurological diseases. BEVs affect the immune system, are involved in the immune response to infections by pathogenic bacteria, and also may influence the immune response in other pathologies. Given the main role of the immune system in different pathologies, its modulation by BEVs could have relevant pathologic or therapeutic effects. Some mechanisms underlying the interaction between BEVs and host cells have been identified, including the interaction between membrane proteins of BEVs and target proteins of host cells, as well as different mechanisms of internalization of BEVs.

BEVs have a role in the communication between the gut and brain in the gut–brain axis. After systemic administration, BEVs reach different brain areas.

Pathologic BEVs affect the permeability of the BBB and induce pathological effects on brain function, including neuroinflammation, anxiety-depression, and cognitive and motor impairment. BEVs also modulate serotonergic and GABAergic neurotransmission in the brain. In addition to BEVs derived from the intestinal microbiota, BEVs from oral microbiota have pathological effects on the brain. For example, BEVs derived from microbiota in oral infections mediate pathological effects in the brain, potentially causing neurodegenerative diseases such as AD.

Conversely, BEVs derived from probiotics may induce beneficial effects. BEVs from probiotics also modulate the immune system and induce beneficial effects in the brain when administered systemically. For example, intravenous injection of BEVs from *Lactobacillus casei* reduces neuroinflammation, restores GABAergic and glutamatergic neurotransmission, and improves motor incoordination and cognitive function in rats with hyperammonemia and minimal hepatic encephalopathy. Similar beneficial effects should be expected in other pathologies associated with neuroinflammation. Main advantages of BEVs as therapeutic tools in neurological and neurodegenerative diseases are their capacity to easily cross the BBB and that they may be easily produced and modified to incorporate different drugs, aiming to enhance the therapeutic potential of beneficial BEVs. Using BEVs as vehicles to deliver drugs into the brain, and even to specific damaged areas, is a strategy that will provide improved therapies for neurological diseases in the near future. The use of BEVs is therefore a promising new therapeutic strategy to treat mental, neurological, and neurodegenerative diseases.

## Future Research

As outlined in this review, there is mounting evidence showing that BEVs, which have the capacity to cross the BBB, exert similar pathological or beneficial effects to their parent bacteria. BEVs from probiotics improve neuroinflammation, neurotransmission, and cognitive and motor function in some neurological diseases, while BEVs from pathologic bacteria induce deleterious effects.

There is great potential for the development of effective therapeutic strategies that exploit the passage of BEVs across the BBB to impact cerebral function. To develop more effective therapeutic approaches using BEVs, there is a series of questions which should be investigated, including the identification of the mechanisms that allow BEVs to cross the BBB and of the components within BEVs that regulate neuroinflammation and neurotransmission, and the underlying mechanisms. These strategies hold considerable translational potential, particularly in the context of improving cognitive and motor function in patients with systemic inflammatory diseases, neurological and neurodegenerative diseases, and in the cognitive and motor impairments associated with aging.

Identifying the mechanisms by which EVs from pathogenic bacteria induce deleterious effects on brain function, neuroinflammation, neurotransmission and cognitive and motor function would allow designing treatments to prevent or reverse these pathological effects in many diseases.

In addition to the mechanisms by which BEVs may cross the BBB, it would also be important to identify the processes and mechanisms by which BEVs may affect the above cerebral processes by acting on the vagus nerve, which involves different mechanisms and could have different therapeutic applications.

These investigations would allow a safe and efficient procedure to treat neuroinflammation, altered neurotransmission, and cognitive and motor function by the modulation of the gut microbiota composition.

Studies on the mechanism involved in the formation and release of BEVs and on the possibility of altering their composition by modifying the culture conditions to enhance their beneficial effects should also be performed.

Future studies involving the use of bioengineering to improve the therapeutic use of BEVs will represent a major advance in improving therapies for neurological diseases, which is currently a field with many deficiencies.

## Additional file:

**Additional Table 1:** *Pathological and beneficial effects of BEVs.*

## Data Availability

*All relevant data are within the paper and its Additional files*.
